# Supplementary material for: Immunoexpression of Relaxin and Its Receptors in Stifle Joints of Dogs with Cranial Cruciate Ligament Disease
Source: Animals (Basel). 2022 Mar 23;12(7):819. doi: 10.3390/ani12070819 (PMC8996950; doi:10.3390/ani12070819)
Supplement: Supplementary file 1 [file animals-12-00819-s001.zip › Sup. mat. Agg GN .pdf]

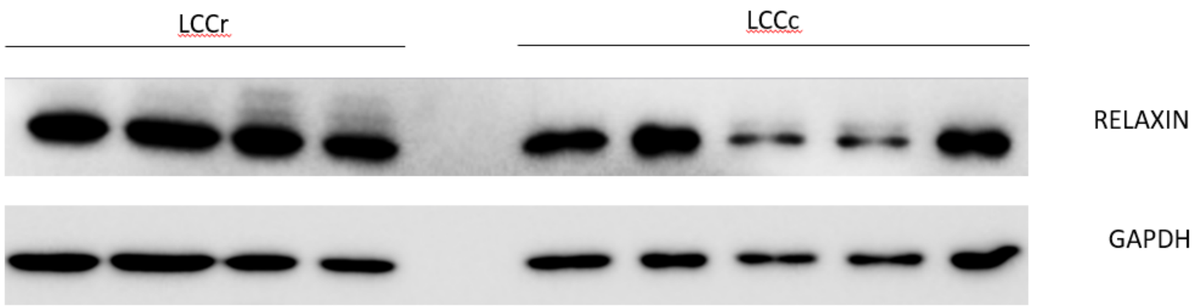

RATIO LCCr REL/GAPDH					RATIO LCCc REL/GAPDH				
0,5486622	0,4896072	0,7295582	0,5656554		0,4433553	0,7884947	0,2874772	0,2714853	0,4778990

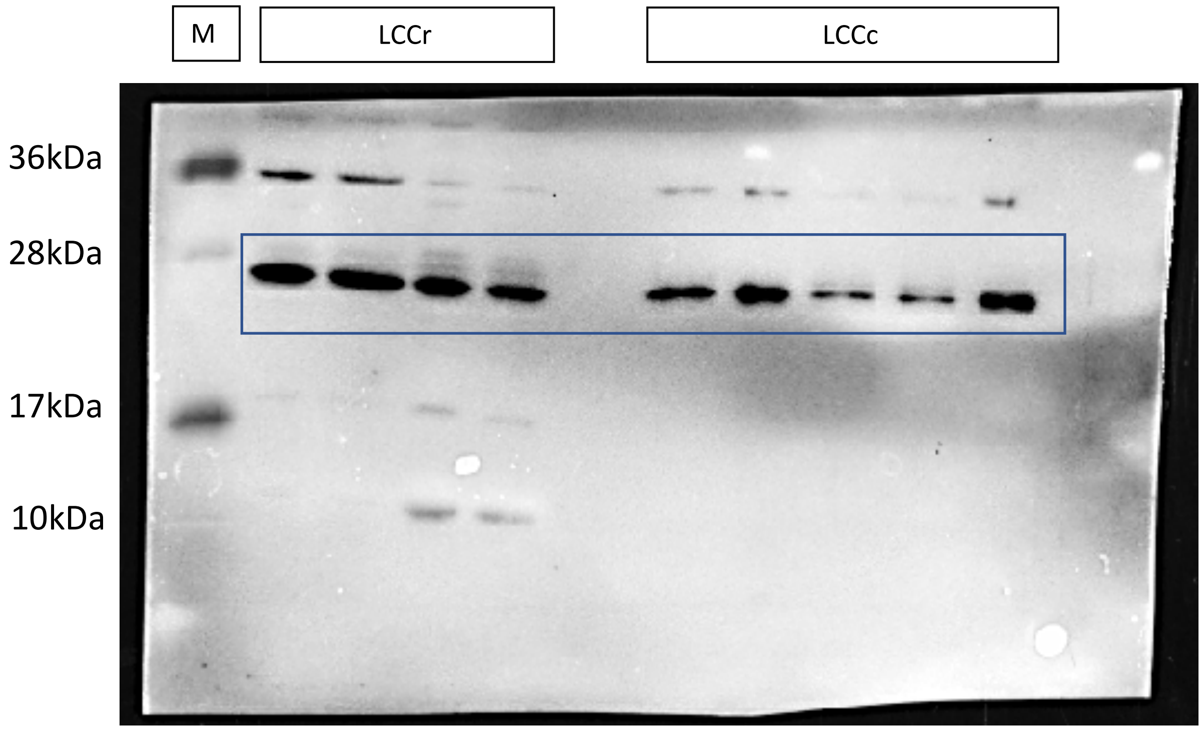

RELAXIN

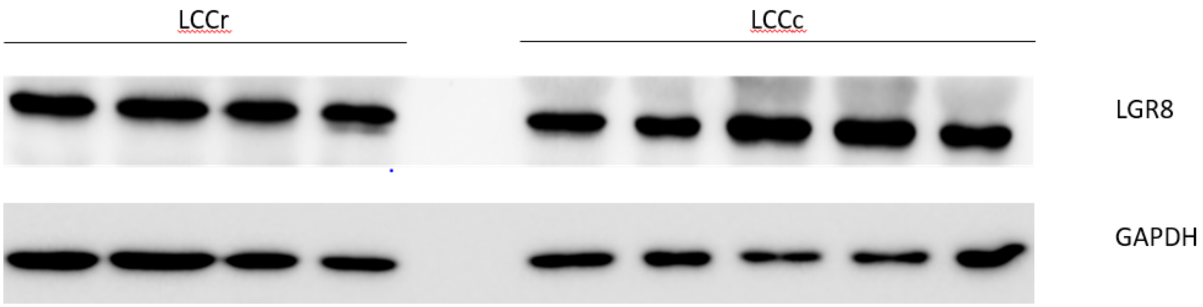

RATIO LCCr LGR8/GAPDH					RATIO LCCc LGR8/GAPDH				
0,769715	0,713414	0,866395	1,119869		1,053313	1,065832	2,649626	2,647485	0,967857

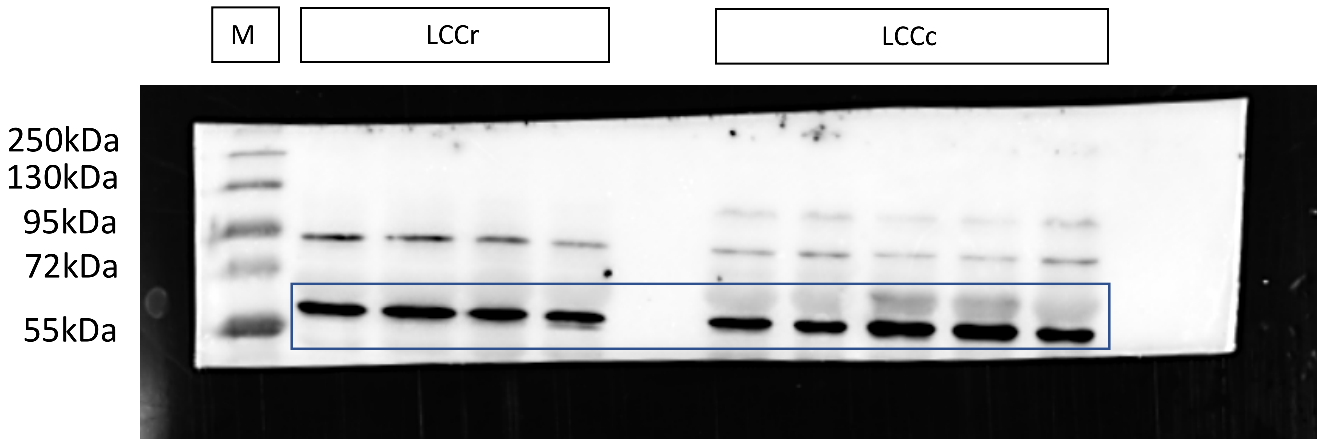
M
LCCr
LCCc
250kDa
130kDa
95kDa
72kDa
55kDa

LGR8

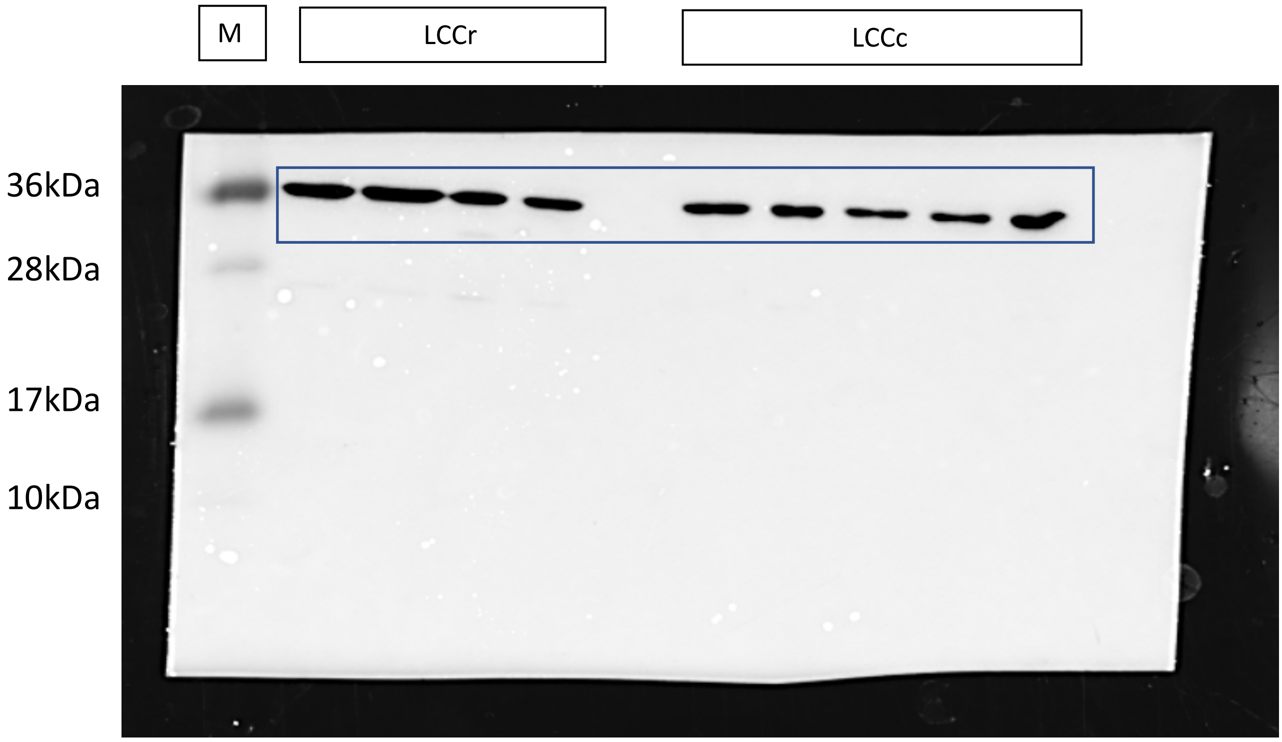
M
LCCr
LCCc
36kDa
28kDa
17kDa
10kDa

GAPDH

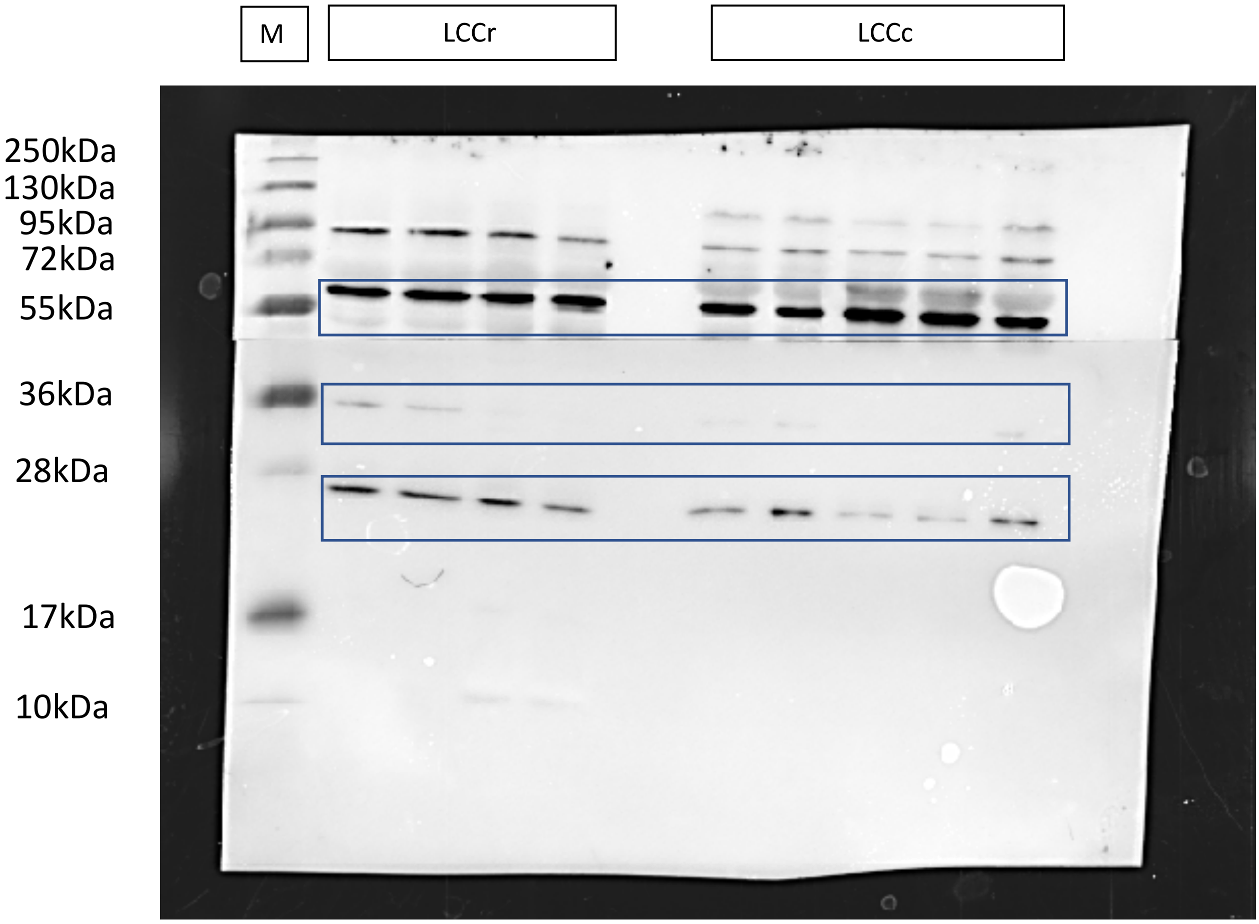
M
LCCr
LCCc
250kDa
130kDa
95kDa
72kDa
55kDa
36kDa
28kDa
17kDa
10kDa

WHOLE BLOT

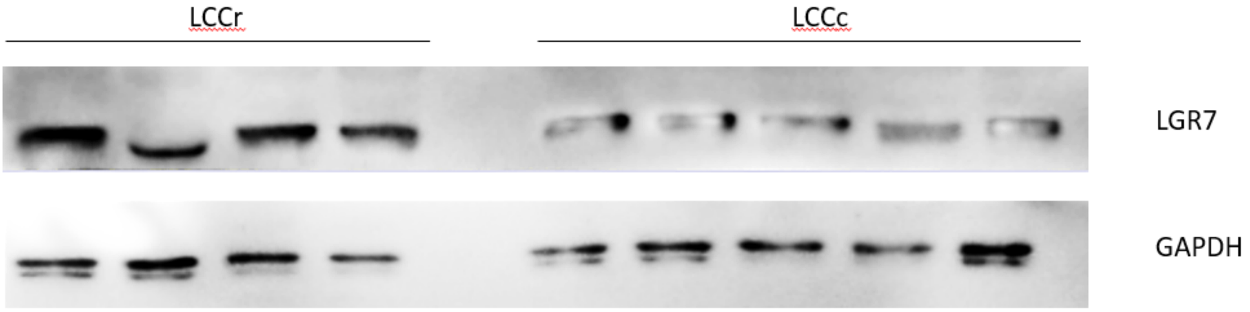

RATIO LCCr LGR7/GAPDH					RATIO LCCc LGR7/GAPDH				
2,43016402	2,4382585	2,7833503	2,6712749		1,0909041	0,894512774	1,006533706	1,545671922	0,7920365

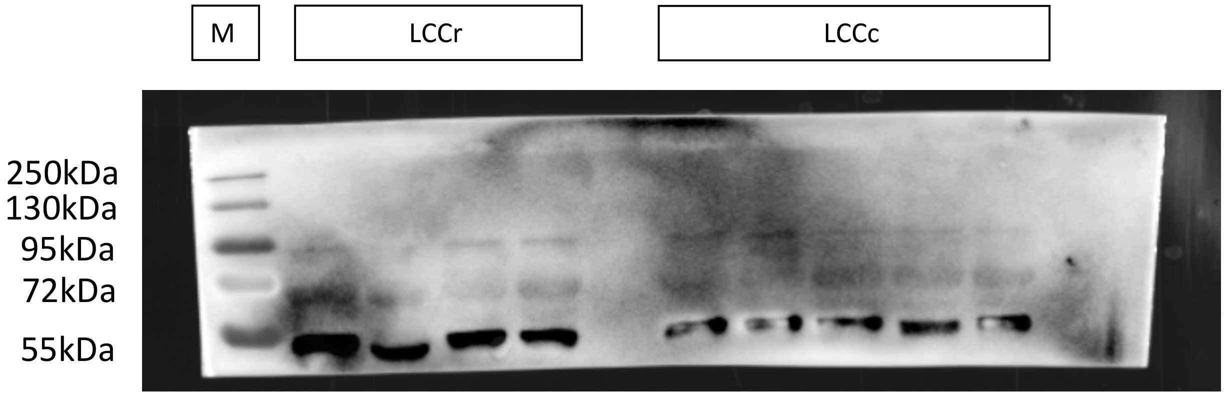

LGR7

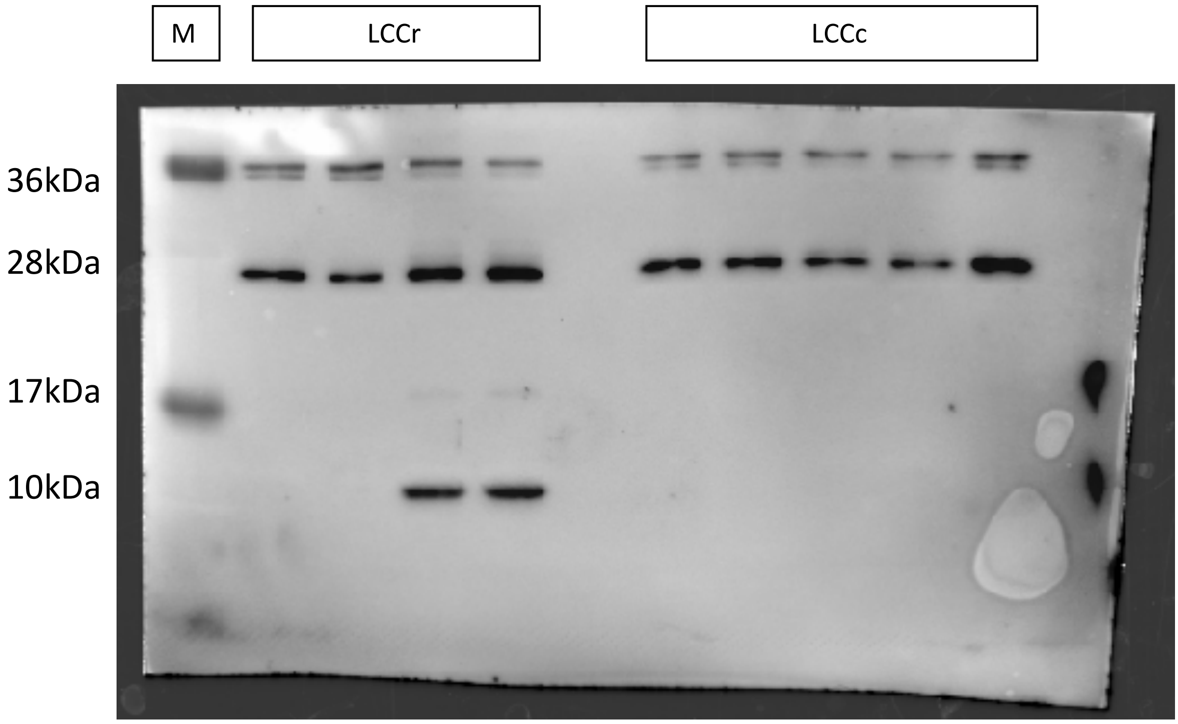

GAPDH

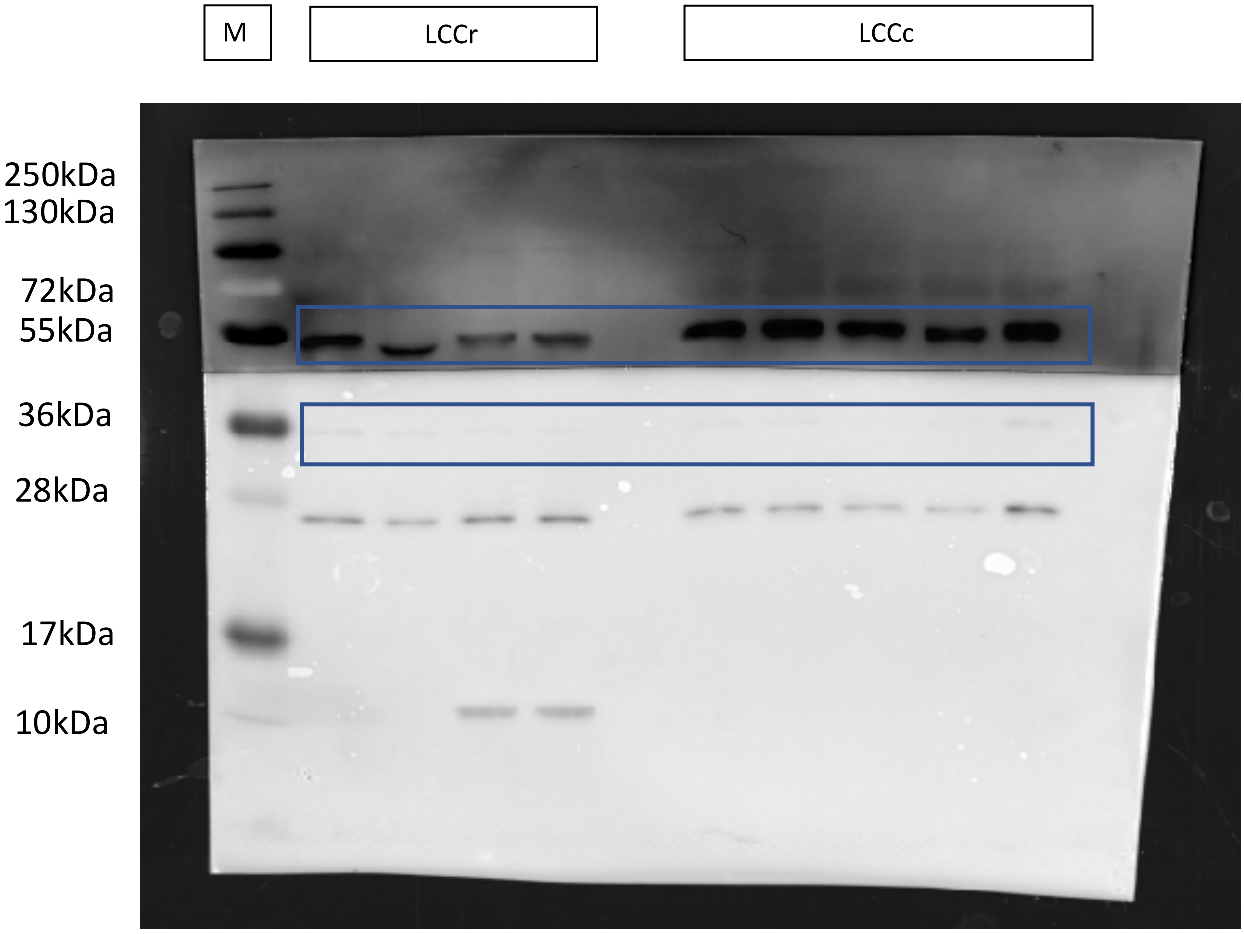
M
LCCr
LCCc
250kDa
130kDa
72kDa
55kDa
36kDa
28kDa
17kDa
10kDa

WHOLE BLOT
