# Supplementary material for: Immunoexpression of Relaxin and Its Receptors in Stifle Joints of Dogs with Cranial Cruciate Ligament Disease
Source: Animals (Basel). 2022 Mar 23;12(7):819. doi: 10.3390/ani12070819 (PMC8996950; doi:10.3390/ani12070819)

Appendix A: Full original blots used for Figure 5a, 5b and 5c. Each blot membrane was cut based on the standard band positions and then incubated with the appropriate antibodies.

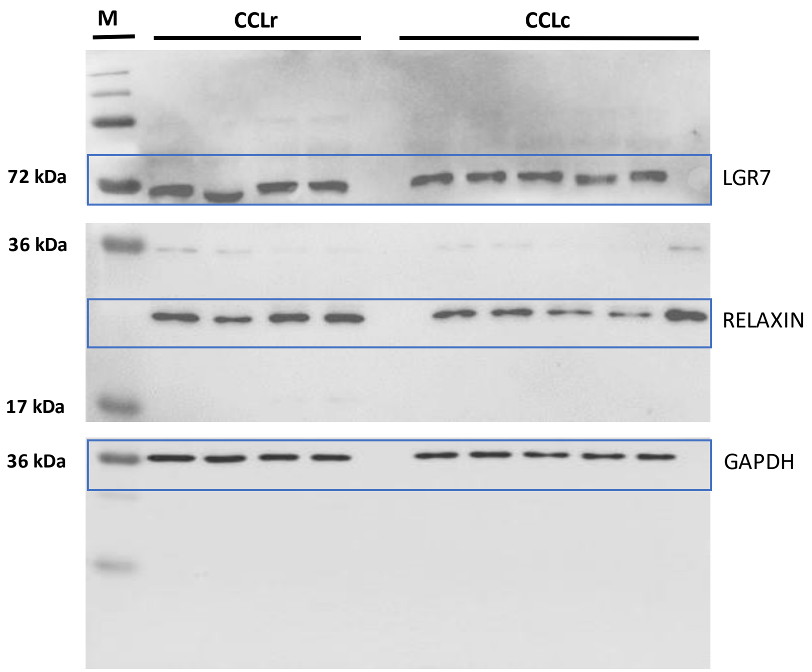

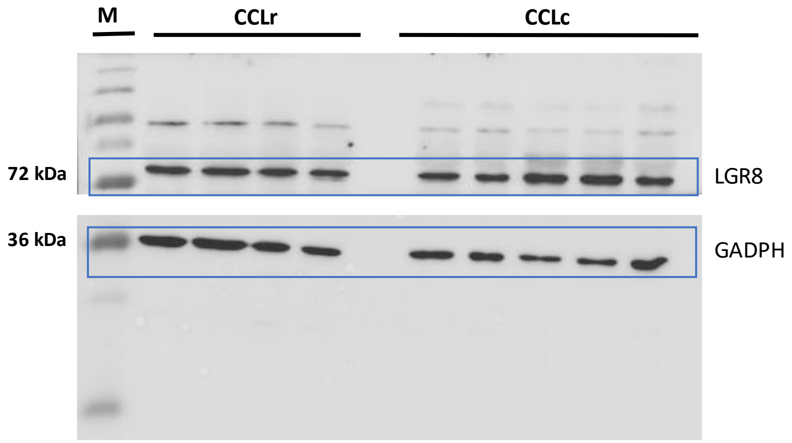

Supplement: Supplementary file 1 [file animals-12-00819-s001.zip › SUPPLEMENTARY MATERIAL.pdf]
